# Trends in symptom severity and complexity in patients undergoing radiation therapy

**DOI:** 10.1186/s12885-025-13587-1

**Published:** 2025-03-04

**Authors:** Demetra Yannitsos, Siwei Qi, Oluwaseun Davies, Linda Watson, Lisa Barbera

**Affiliations:** 1 Department of Oncology, University of Calgary, Calgary, Canada; 2 Cancer Care Alberta, Alberta Health Services, Calgary, Canada; 3 Tom Baker Cancer Centre, Calgary, Canada

## Abstract

Symptom severity and complexity have considerable impact on a patient’s cancer care journey. This study describes symptom scores of radiotherapy patients across their radiotherapy care trajectory and factors associated with symptom complexity. Patients who received radiotherapy at a single tertiary cancer center, who also completed at least one symptom-reporting questionnaire, the Edmonton Symptom Assessment Scale– Revised (ESAS-r) between October 1, 2019 and April 1, 2020 were included in this retrospective analysis. Symptom assessment time points were pre-treatment, start and end of radiation treatment and post-treatment follow-up. Mean ESAS-r scores for individual symptoms were descriptively analyzed by assessment timing and tumour group. We calculated a symptom complexity score for each ESAS-r measurement, using a validated algorithm, and assigned overall symptom complexity as low, moderate or severe. We modelled the association between assessment timing, and tumor group, with symptom complexity using Generalized Estimating Equations (GEE). The study cohort consisted of 1,632 patients who completed 2,519 ESAS-r questionnaires. Patients with lung and H&N cancers reported higher mean symptom scores compared to other tumour groups. Patients at the start of treatment had significantly lower odds of having a more severe symptom complexity, compared with patients pre-treatment (OR = 0.77, 95% CI = 0.64–0.93). Patients with H&N and lung cancer and patients prior to starting radiation may benefit most from increased symptom support and management.

**Supplementary Information:**

The online version contains supplementary material available at 10.1186/s12885-025-13587-1.

## Introduction

Oncology patients receiving treatment experience a dynamic range of symptoms [1]. Common symptoms include psychological symptoms such as anxiety and depression, as well as physical symptoms such as pain, appetite loss and fatigue [2–4]. Understanding symptom trajectory across the continuum of care is crucial for providing optimal care and improving quality of life.

Patient-reported outcomes measures (PROMs) enable the capture of patient’s perspective on their symptoms. Routine collection of PROMs has been shown to identify unmet symptom needs, improve communication, improve treatment adherence, reduce utilization of acute care services, and improve survival [5–9]. Within CancerCare Alberta (CCA), a provincial ambulatory program, PROMs, including the Edmonton Symptom Assessment Scale– Revised (ESAS-r), are routinely incorporated into clinical workflows and are administered to all oncology patients receiving ambulatory care [10]. Typically, ESAS-r is collected pre-treatment, at the start and end of radiation therapy, and post treatment.

In addition to assessing and monitoring individual ESAS-r scores, symptom complexity scores have become increasingly utilized within CancerCare Alberta to provide healthcare professionals with a quick overview of a completed ESAS-r form, by using a validated algorithm to assign each encounter with a low, moderate or severe score [11]. Complexity scores provide an effective way of flagging highly complex patients who may require additional time and clinical support [11]. 

There is limited research reporting on symptom score trends in a radiation-specific cohort across various tumour groups. Previous studies have included general cancer patients in their samples (not radiation specific) [2–4], have looked at symptoms cross-sectionally [2], temporally over months [3] or during the end of life specifically [4]. Understanding symptom scores and complexity across the radiation treatment trajectory can help clinicians understand and better support patients, especially with data collected at routine appointments where patients see their oncologist and radiation team. This study describes ESAS-r symptoms and complexity scores overtime in patients receiving radiation treatment at a large tertiary ambulatory cancer care centre and compares them across tumour groups and time points.

## Methods

### Study design

This retrospective observational study was conducted using several sources of linked electronic healthcare data. The dataset used was part of a larger study investigating symptom burden in oncology patients that received ethics approval from the Health Research Ethics Board of Alberta’s Cancer Committee (HREBA.CC-20-0022).

### Cohort ascertainment

The study cohort was comprised of patients in Alberta who were 18 years of age and older with any cancer diagnosis, who had at least one radiation therapy appointment at the Tom Baker Cancer Centre (TBCC) between October 1, 2019 and April 1, 2020. To be eligible for inclusion, patients also had to have completed at least one ESAS-r questionnaire within this timeframe at any one of the following time points: radiation consultation (pre-treatment), first and last radiation treatment review and first follow up post radiation treatment. Patients whose ESAS-r was not completed within 2 days of the appointment date were excluded.

### Study setting

At the time of this study, ESAS-r was administered in clinic, on paper. Responses were reviewed by the clinical team and nurses manually documented the scores in the EMR. Historical scores were available on a digital dashboard.

### Data sources and variables

The study utilized administrative data from the Alberta Cancer Registry (ACR), clinical data from CCA’s electronic medical record (EMR), and from the Canadian Institute for Health Information (CIHI) Discharge Abstract Database (DAD). Data linkage was deterministically achieved through a unique provincial health care number assigned to each patient as part of the ACR’s process at the time.

We collected age, sex, tumour group, and rurality index from the ACR database; Charlson Comorbidity Index (CCI) from the DAD database; and appointment types/dates from the EMR. A modified version of the CCI was used which excluded cancer as a condition so that this did not contribute to the index score, as all participants in this study had a cancer diagnosis [12, 13]. Rurality index for each patient was assigned based on the postal code of their most recent residence, using a seven-level index created by Alberta Health Services (AHS), and assigned to one of three groups for analysis: urban (core of city), and metro (the wider area around an urban centre), rural (areas outside metro areas) [14]. Tumour groups were defined as breast, gastrointestinal (GI), genitourinary (GU), gynecology (gyne), hematology (hem), head and neck (H&N), lung and other.

Time points were defined relative to the radiation treatment journey: pre-treatment (initial radiation consultation appointment, start of radiation (first review appointment scheduled for the patient to see their radiation oncologist within the first week of starting treatment), end of radiation (last review appointment scheduled for the patient to see their radiation oncologist within the final week of treatment) and post radiation treatment (first follow up appointment after treatment completion). Time intervals between first and last review could vary from a single visit, to several weeks. The first follow up typically occurred 3 months post radiation treatment, however the first radiation treatment follow up was always chosen.

### Outcome

*The ESAS-r* is a 9-item PROM which measures prevalent symptoms experienced by patients with cancer [15–17]. Patients rate each symptom on a severity scale from 0 to 10, with 10 indicating the highest severity. The 9 ESAS-r symptoms include pain, tiredness, drowsiness, nausea, lack of appetite, shortness of breath, depression, anxiety and wellbeing.

*The symptom complexity* score, derived from ESAS-r, utilizes a validated algorithm which considers the unique combination of symptoms and concerns the patient has identified [11]. It rates the self-reported severity of symptoms indicated at a single visit and assigns a symptom complexity score (low, moderate, or severe) for the encounter (see Additional File 1 for the algorithm used to assign symptom complexity scores).

### Statistical analyses

Demographic data and symptom outcomes were summarized using descriptive statistics. Mean symptom scores for individual ESAS-r symptoms were summarized by appointment timing and tumour group. Scores described by tumour groups included all time points, meaning there could be multiple observations per patient.

P-values were also calculated for individual symptom scores and symptom complexity scores across time points and tumour groups. The main effect of time on outcomes was examined by applying Generalized Estimating Equations (GEE) to account for repeated measures. To examine the main effect of tumour groups on outcomes, GEE was applied with patient ID as the clustering variable to account for within patient correlation.

The association of appointment timing and tumor group with symptom complexity was modelled using GEE, with the outcomes as ordinal (low, moderate, and high). We used the GEE approach to consider within-subjects variability and account for the correlated data resulting from repeated measurements across different time points and multiple observations of the same individual [18]. These methods of analysis have been utilized in other similar studies [19]. 

Data were exported into SPSS Version 25.0 (Chicago, IL, USA) and SAS statistical software Version 9.4 (SAS Institute, Cary, NC) for analysis and statistical significance was set a priori at *p* < 0.05.

## Results

### Study sample

Table 1 presents the baseline demographic information for the full study cohort (*N* = 1,632). The mean age was 63.4 years, and 935 participants (57.3%) were male. The majority (81.5%) of the cohort lived in a metro area. The most common tumour group was breast (31.1%), followed by genitourinary (17.4) and lung (13.1%). A small portion of the cohort (13.4%) had a Charlson Comorbidity Index (CCI) score at or above 1.

**Table 1:** Cohort characteristics

	*n*	%
Age		
Mean (SD)	63.4 (12.7)	
Sex		
Female Male	697 935	42.7 57.3
Rurality Index		
Metro Urban Rural	1,311 82 216	81.5 5.1 13.4
Tumour Group		
Breast Gastrointestinal Genitourinary Gynecology Hematology Head & Neck Lung Other*	508 176 284 107 69 109 213 166	31.1 10.8 17.4 6.6 4.2 6.7 13.1 10.2
CCI		
0 ≥1	1,414 218	86.6 13.4
Days between first and last review		
Mean (SD)	29.0 (25.7)	

Other included: central nervous system (CNS), endocrine, melanoma, no melanoma skin, other malignant and sarcoma

Note: SD = standard deviation, CCI = Charlson comorbidity index

Within the six-month study period, 1,632 patients completed 2,519 ESAS-r questionnaires within 2 days of their appointment date. Of the 2,519 questionnaires, 1,001 (39.7%) were collected at consult, and 727 (28.9%), 583 (23.1%) and 208 (8.3%) were collected at the start of treatment, end of treatment and follow-up, respectively.

Mean Individual ESAS-r Symptoms.

**Fig. 1:**
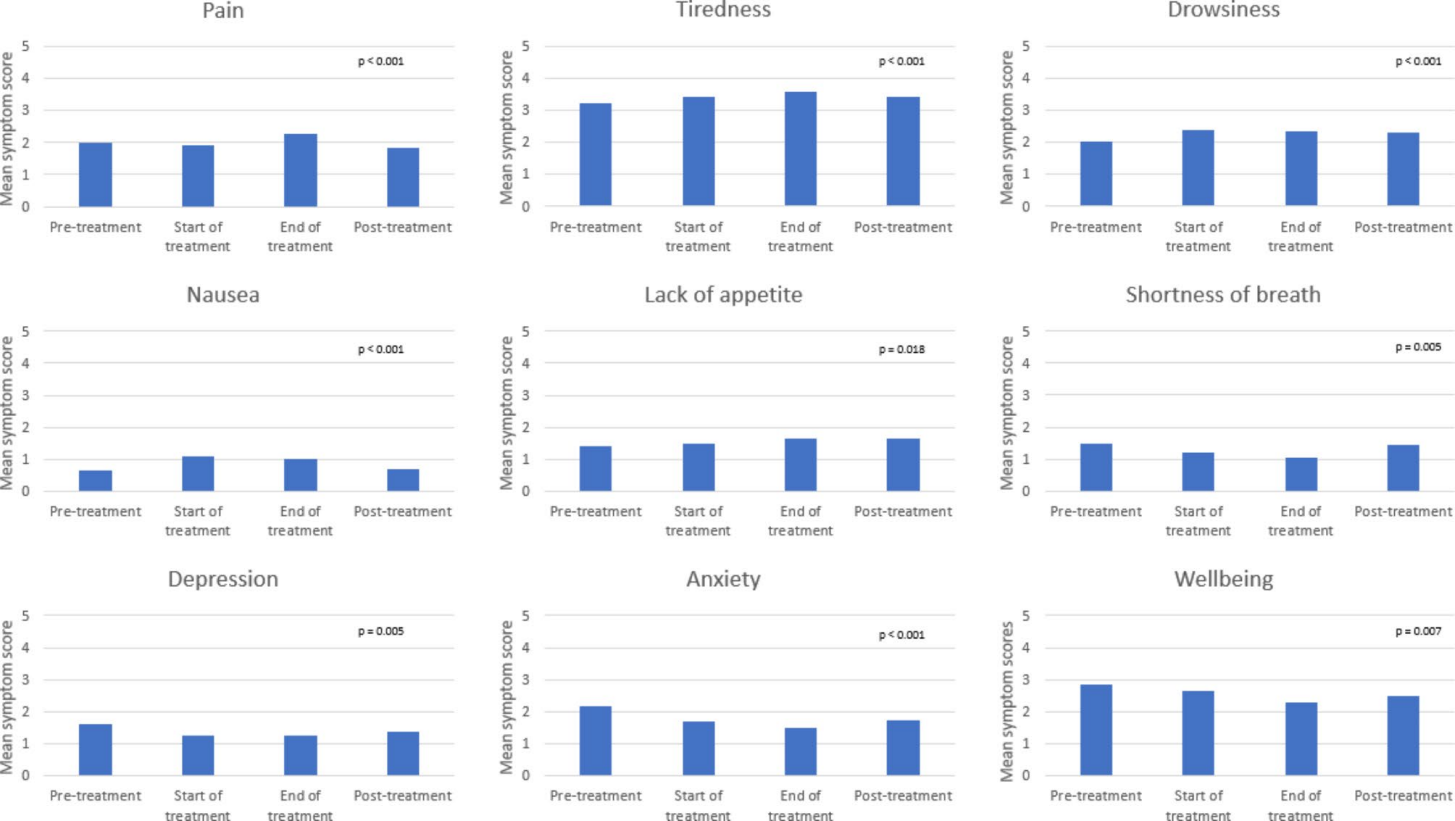
Mean ESAS-r symptom scores for individual symptoms at radiation time points. Note: each symptom is scored 0–10 (10 being most severe)

Mean ESAS-r scores by individual symptom were compared across time point (see Fig. 1). Symptoms with highest (most severe) mean scores pre-treatment included well-being (mean = 2.85), anxiety (2.19), shortness of breath (1.49) and depression (1.60). At the end of treatment, tiredness (mean = 3.55), pain (2.26) and lack of appetite (1.66) were reported highest across time points. Individual symptom scores were significant across time points (p-values < 0.05 for all symptoms) (Fig. 1).

**Fig. 2:**
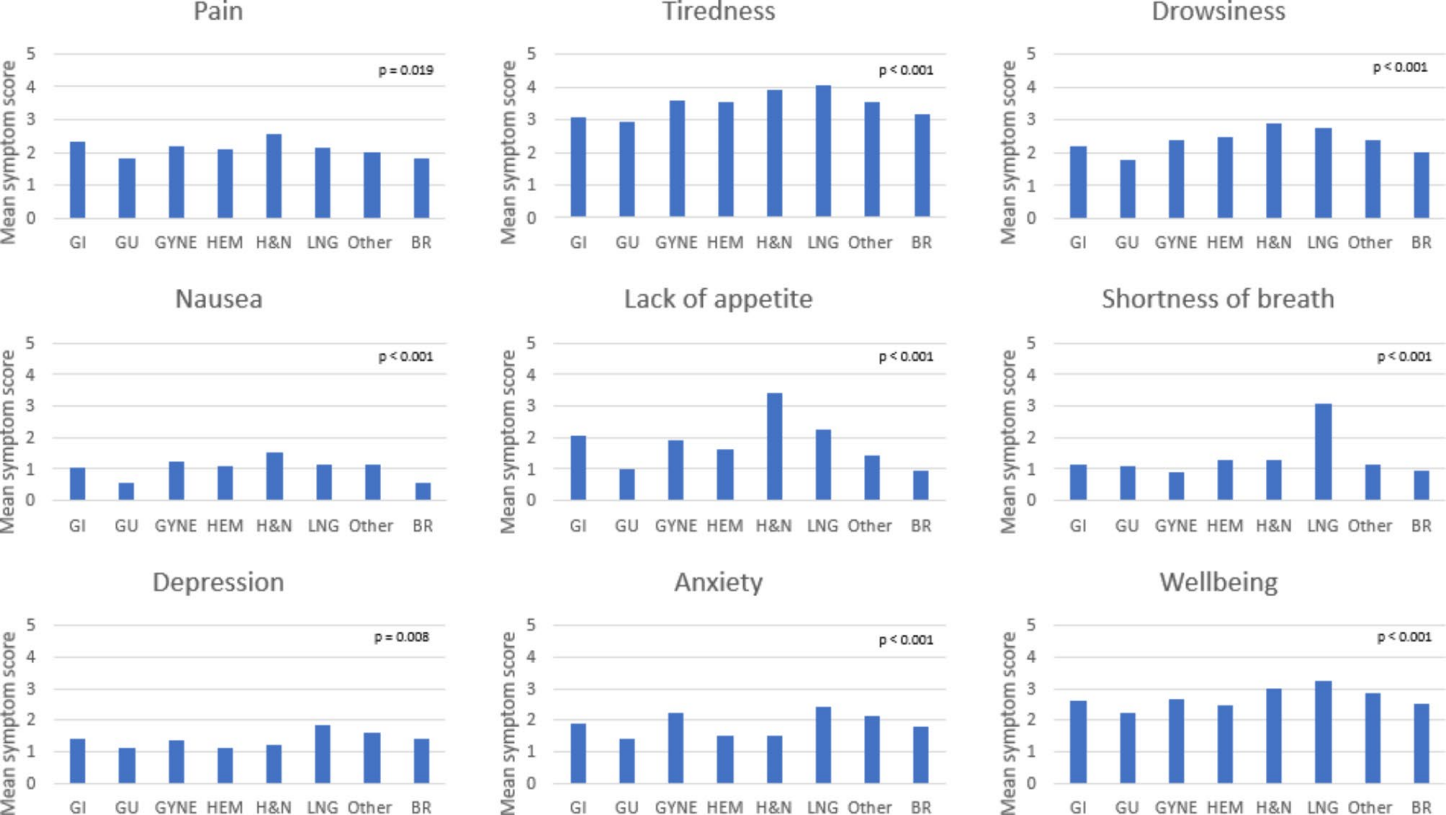
Mean ESAS-r symptom scores across tumour groups. Note: each symptom is scored 0–10 (10 being most severe). BR = breast; GI = gastrointestinal; GU = genitourinary; GYNE = gynecology; HEM = hematology; H&N = head and neck; LNG = lung

Mean ESAS-r scores for individual symptoms were also compared across tumour groups (Fig. 2). H&N patients recorded the highest mean scores for 4/9 symptoms, including lack of appetite (3.40), drowsiness (2.87), pain (2.55) and nausea (1.53). Lung patients recorded the highest mean scores for 5/9 symptoms, including tiredness (4.06), poor well-being (3.24), shortness of breath (3.07), anxiety (2.44) and depression (1.86). All individual symptoms were significant across tumour groups (p-values < 0.05) (Fig. 2).

### Descriptive statistics-symptom complexity

The proportion of low, moderate and high symptom complexity scores of the sample are shown in Table 2. Across time points, the highest proportion of high symptom complexity scores was demonstrated pre-treatment (19.3%) and lowest proportion at the start of treatment (13.2%), although not statistically significant across time points (p-0.178) (Table 2)Across tumour groups (Table 3), Lung and H&N reported the highest proportions of high symptom complexity scores (25.8% and 23.9%, respectively), with smallest proportions in GU and Breast (13.8% and 12.7%, respectively). Complexity scores across tumour groups were significant (*p* < 0.001) (Table 3).

**Table 2:** Symptom complexity across radiation timepoints

	Pre-treatment (*n* = 1,001)	Start of treatment (*n* = 727)	End of treatment (*n* = 583)	Post-treatment (*n* = 208)	*p*-value
Symptom complexity	n (%)	n (%)	n (%)	n (%)	0.178
Low	622 (62.1%)	475 (65.3%)	382 (65.5%)	134 (64.4%)	
Moderate	186 (18.6%)	156 (21.5%)	112 (19.2%)	40 (19.2%)	
High	193 (19.3%)	96 (13.2%)	89 (15.3%)	34 (16.3%)	

Note: independent variable is timepoint when testing for significance across symptom complexity scores

**Table 3:** Symptom complexity by tumour group

	BR (*n* = 898)	GI (*n* = 272)	GU (*n* = 383)	GYNE (*n* = 175)	HEM (*n* = 111)	H&N (*n* = 155)	LNG (*n* = 299)	Other (*n* = 226)	*p*-value
Symptom complexity	n (%)	n (%)	n (%)	n (%)	n (%)	n (%)	n (%)	n (%)	< 0.001
Low Moderate High	618 (68.8%) 166 (18.5%) 114 (12.7%)	171 (62.9%) 52 (19.1%) 49 (18.0%)	277 (72.3%) 53 (13.8%) 53 (13.8%)	106 (60.6%) 41 (23.4%) 28 (16.0%)	75 (67.6%) 18 (16.2%) 18 (16.2%)	83 (53.5%) 35 (22.6%) 37 (23.9%)	147 (49.2%) 75 (25.1%) 77 (25.8%)	136 (60.2%) 54 (23.9%) 36 (15.9%)	

Note: BR = breast; GI = gastrointestinal; GU = genitourinary; GYNE = gynecology; HEM = hematology; H&N = head and neck; LNG = lung

Note: independent variable is timepoint when testing for significance across symptom complexity scores

### Factors associated with Symptom Complexity scores

We observed a significant association between appointment timing and symptom complexity after correcting for baseline covariates. Patients at the start of treatment were less likely to have a higher (more severe) symptom complexity score, compared with patients pre-treatment (OR = 0.77, 95% CI = 0.64–0.93). Patients at the end of treatment and post treatment also had lower odds of having a more severe complexity score, however not at a significant level (Table 4).

We also observed a significant association between tumour group and symptom complexity after correcting for baseline covariates. Compared to patients with breast cancer, patients with GI, H&N, lung and other cancers were significantly more likely to have higher symptom complexity scores (ORs ranged from 1.65 to 2.77). Differences between the patients with breast cancer and GU, gynecology, and hematology cancers were not significant. Details are shown in Table 4.

**Table 4:** GEE results of parameters associated with an odds ratio of having a higher symptom complexity score (*n* = 1632)

	OR (95% CI)	*p*
Age	0.96 (0.99-1.00)	0.211
Sex		
Male* Female	1 **0.65 (0.49–0.86)**	**0.002**
Charlson comorbidity index		
CCI ≥ 1* CCI = 0	1 **0.66 (0.51–0.87)**	**0.003**
Rurality		
Rural* Metro Urban	1 1.07 (0.80-1.42) 1.16 (0.71–1.91)	0.665 0.548
Appointment type		
Pre-treatment* Start of treatment End of treatment Post-treatment	1 **0.77 (0.64–0.93)** 0.84 (0.67–1.02) 0.81 (0.60–1.10)	**0.005** 0.079 0.171
Tumour Group		
Breast* Gastrointestinal Genitourinary Gynecology Hematology Head & Neck Lung Other	1 **1.65 (1.14–2.40)** 1.31 (0.86–1.99) 1.38 (0.92–2.07) 1.47 (0.85–2.54) **2.77 (1.80–4.26)** **2.73 (1.93–3.85)** **1.70 (1.16–2.48)**	**0.008** 0.207 0.125 0.166 **0.000** **0.000** **0.006**

* Reference group

Note: OR = odds ratio, CI = confidence interval

## Discussion

This study describes ESAS-r mean symptom scores and symptom complexity trends in a radiation-oncology specific cohort. Across tumour groups, patients with H&N and lung cancers report higher symptom scores and were most likely to have high symptom complexity. Symptom complexity scores were highest pre-treatment, with well-being and anxiety having the highest mean scores at this time point. These results highlight tumour groups that may benefit most from enhancing symptom support and management within the radiation department and that symptoms tend to be worst prior to starting radiation.

We found for individual mean symptom scores, anxiety, depression and wellbeing were reported highest prior to treatment. Other studies have also found anxiety and depression scores elevated at the beginning of patients’ cancer treatment trajectory [3, 20, 21]. Bubis et al. found lower odds of elevated anxiety and depression scores with each month relative to the month of diagnosis [3]. Encouraging patients to access available resources, and educating patients on various support options early may benefit the patient. Providing support between referral and consultation may be particularly effective. The most cited barrier to oncology patients accessing supportive services is a lack of awareness of available supports, and a lack of referrals from their physicians [22]; however, patients are often overwhelmed at consultation. Even when healthcare providers encourage discussions of available supportive care services, many patients cannot retain or remember those details afterwards. An additional education-based appointment scheduled shortly after the patient’s consultation could potentially help to address these issues.

Our results show mean physical symptom scores were generally higher at the end of radiation compared to pre-treatment, which would likely be due to treatment-related toxicities [23, 24]. Mean symptom scores for pain, tiredness and lack of appetite peaked at the end of treatment, similar to other studies [3, 23]. Although increased symptom severity is in part unavoidable due to the radiation treatment, earlier interventions and referrals to allied health could play a role in decreasing overall symptom severity. Implementing a tumour-specific PROM weekly during radiotherapy has been found to be feasible and accepted, with the potential to help physicians identify problematic symptoms earlier on in their patients’ treatment trajectory [25–27]. 

One study reported mean ESAS symptom scores for radiation therapy patients at radiation consultations and at the end of treatment [23]. In curative-intent radiation patients, the top 3 (most severe) mean ESAS symptom scores pre-treatment matched our study, including well-being (3.30 vs. 2.85), tiredness (2.92 vs. 3.20) and anxiety (2.72 vs. 2.19), respectively. Mean ESAS scores measured at the end of radiation treatment were highest for tiredness (4.46), well-being (3.81) and appetite (3.48) which varied slightly from our study: tiredness (3.60), drowsiness (2.35), and wellbeing (2.28).

Mean symptom scores across tumour groups show patients with H&N and lung cancers reporting the highest mean scores. Patients with H&N and lung, as well as other and GI cancers, had greater odds of a worse symptom complexity score, compared to patients with breast cancer. Greater symptom burden in patients with H&N and lung cancers has been previously reported in both tumour groups [3, 28.^29^] and lung cancer specifically [3, 30, 31]. Although patients with lung cancer can present with particularly complex symptom profiles, many do to not engage with supportive resources or services [22, 32, 33]. In a US study of breast, lung, GI and other tumour groups, results indicated that patients with lung cancer were half as likely to access supportive care and palliative care services compared to the other tumour groups [22]. However, when patients with lung cancer do engage in interdisciplinary supportive care they do benefit [34, 35]. Patients with lung cancer may be a particularly important group to help improve symptom management, and may require additional clinical time and resources including promoting uptake of supportive resources.

We found patient characteristics significantly associated with symptom complexity. Individuals with comorbidities were more likely to have a higher symptom complexity score. This is unsurprising and has been found in other oncology studies [2, 3]. We also found females had greater odds of a higher symptom complexity score compared to males. Females have reported more severe cancer symptoms in other studies [2, 3], however the literature remains inconsistent [36–38]. 

There were limitations to our study. This study utilized real-world data, and therefore not all patients filled out the PROs at all four timepoints. ESAS-r is routinely collected at our institution, but completion is voluntary, which may have over or under-represented certain sub-populations. Those more ill may be less likely or able to complete ESAS-r compared to those who are well, therefore potentially underestimating symptom burden. ESAS-r is also a generic PRO, and is missing site-specific symptoms that would provide further insights into tumour or radiation specific symptom profiles. Our dataset included patients who received radiation therapy, however our dataset was limited and did not allow us to distinguish between patients receiving radiation alone vs. concurrent chemo-radiation, details of radiation site, or treatment intent. Further, PROs are becoming increasingly adopted into oncology clinical practice, though patient adherence to PRO completion overtime remains an issue for many institutions [39], as seen in our sample, therefore limiting our sample size and ability to analyze trends in greater detail. However, we are still able to provide ESAS-r data aggregated at specific points over time anchored to key clinical events, helping to identify when patients need increased support.

## Conclusion

Our results highlight significant differences in symptom experiences at different time points and in certain tumour groups. These represent targets for additional supportive care. Cancer care organizations and radiation departments may apply these findings to build patient-centered quality improvement initiatives tailored to specific time points in care.

## Electronic supplementary material

Below is the link to the electronic supplementary material.

Supplementary Material 1: Additional File 1. Symptom Complexity Score. Figure shows the algorithm used to derive the symptom complexity score.

## Data Availability

No datasets were generated or analysed during the current study.
