# Supplementary figures and images for: Trends in symptom severity and complexity in patients undergoing radiation therapy
Source: BMC Cancer. 2025 Mar 4;25:390. doi: 10.1186/s12885-025-13587-1 (PMC11877897; doi:10.1186/s12885-025-13587-1)

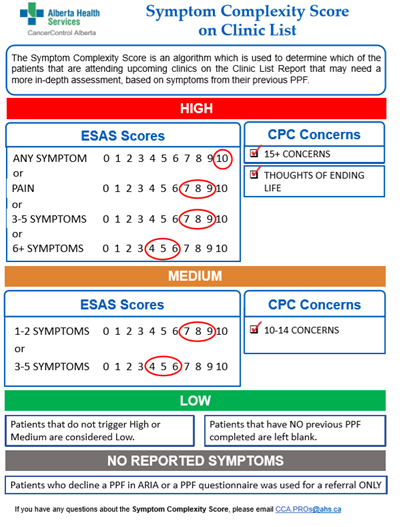

Supplement: Supplementary file 1 — Supplementary Material 1: Additional File 1. Symptom Complexity Score. Figure shows the algorithm used to derive the symptom complexity score. [file 12885_2025_13587_MOESM1_ESM.png]
